# Proteomic analysis of purified Newcastle disease virus particles

**DOI:** 10.1186/1477-5956-10-32

**Published:** 2012-05-09

**Authors:** Xiangpeng Ren, Chunyi Xue, Qingming Kong, Chengwen Zhang, Yingzuo Bi, Yongchang Cao

**Affiliations:** 1 School of Environmental Science and Public Health, Wenzhou Medical College, Wenzhou, 325035, Peoples Republic of China; 2 State Key Laboratory of Biocontrol, School of Life Sciences, Sun Yat-sen University, Guangzhou, 510006, Peoples Republic of China; 3 College of Animal Science, South China Agricultural University, Guangzhou, 510642, Peoples Republic of China

## Abstract

**Background:**

Newcastle disease virus (NDV) is an enveloped RNA virus, bearing severe economic losses to the poultry industry worldwide. Previous virion proteomic studies have shown that enveloped viruses carry multiple host cellular proteins both internally and externally during their life cycle. To address whether it also occurred during NDV infection, we performed a comprehensive proteomic analysis of highly purified NDV La Sota strain particles.

**Results:**

In addition to five viral structural proteins, we detected thirty cellular proteins associated with purified NDV La Sota particles. The identified cellular proteins comprised several functional categories, including cytoskeleton proteins, annexins, molecular chaperones, chromatin modifying proteins, enzymes-binding proteins, calcium-binding proteins and signal transduction-associated proteins. Among these, three host proteins have not been previously reported in virions of other virus families, including two signal transduction-associated proteins (syntenin and Ras small GTPase) and one tumor-associated protein (tumor protein D52). The presence of five selected cellular proteins (i.e., β-actin, tubulin, annexin A2, heat shock protein Hsp90 and ezrin) associated with the purified NDV particles was validated by Western blot or immunogold labeling assays.

**Conclusions:**

The current study presented the first standard proteomic profile of NDV. The results demonstrated the incorporation of cellular proteins in NDV particles, which provides valuable information for elucidating viral infection and pathogenesis.

## Background

Newcastle disease (ND) is a contagious fatal viral disease affecting most species of birds, which was classified as a list A infectious disease by the World Organization for Animal Health. Newcastle disease virus (NDV) as the etiological agent for ND is a nonsegmented single-stranded negative sense RNA virus that belongs to the genus *Avulavirus* within the *Paramyxoviridae* family [1]. NDV is endemic to many countries, most notably in domestic poultry due to their high susceptibility, and has caused tremendous economic consequences to the poultry industry throughout the world. NDV virion contains at least six structural proteins, Haemagglutinin Neuraminidase (HN), Fusion protein (F), Matrix protein (M), Nucleocapsid protein (NP), Phosphate protein (P) and Large protein (L) [2-5]. HN and F are the two surface glycoproteins of viral envelope membrane, whereas NP、P、L and membrane-associated M are inner components of NDV virions [2-5]. F protein, which is considered to be the key virulence determinant of the virus, mediates the fusion process between viruses and cell membranes [6-8]. HN is a multifunctional virion protein, which plays roles in helping membrane fusion, cell tropism determination and viral pathogenicity [9-11]. M lies beneath the viral membrane and surrounds the ribonucleoprotein (RNP) complex [12]. The RNP complex consists of the viral RNA coated with NP and bound by the polymerase complex that contains P and L [13].

It has been reported that many host proteins might be packaged into the enveloped virions along with the viral components during the virus life cycle, but the role of these cellular proteins in viral infection are not fully understood [14,15]. Identification of the protein composition of the infectious virions has important implications for understanding the interaction of viruses with host cells, which provides valuable information for elucidating viral replication, tropism and virulence [16].

Due to enhanced proteomic techniques based on two-dimensional gel electrophoresis (2-DE) separation and Mass spectrometry (MS) combined with database searching for identification, virion proteomics (the protein composition of the purified virus particles) becomes a useful tool in global evaluation of interaction between viruses and their hosts through identifying cellular proteins in virions [16]. Numerous host proteins have been found that incorporate into the membranes or inside the envelopes of the virions using virion proteomic approaches. Herpes virus, an enveloped DNA virus which is a leading cause of human viral diseases, is currently the best studied virus group. Among this group are human cytomegalovirus (HCMV) [17], murine cytomegalovirus (MCMV) [18], Epstein-Barr virus (EBV) [19], Kaposi’s sarcoma-associated herpesvirus (KSHV) [20,21], rhesus monkey rhadinovirus (RRV) [22], Marek’s disease virus (MDV) [23] and murine gammaherpesvirus 68 (MHV68) [24]. Moreover, virion proteomics have been performed for other enveloped DNA viruses, such as vaccinia virus (VV) [25,26], gigantic mimivirus [27], White spot syndrome virus (WSSV) [28,29] and Singapore grouper iridovirus (SGIV) [30].

Compared with enveloped DNA viruses, only a few enveloped RNA viruses have been analyzed by virion proteomics, potentially because of the relatively simpler structures and lower number of proteins encoded by RNA viruses. The most well studied RNA virus is retrovirus human immunodeficiency virus (HIV). Proteomic analysis revealed that HIV virions contain a high number of host cell proteins [31,32]. Severe acute respiratory syndrome (SARS) coronavirus has also been analyzed by virion proteomics [33,34]. In addition, 36 host-encoded cellular proteins have been found to incorporate into influenza virus (IV) virions [35]. Other enveloped RNA viruses which have been proteomically analyzed were vesicular stomatitis virus (VSV) [36], infectious bronchitis virus (IBV) [37] and porcine reproductive and respiratory syndrome virus (PRRSV) [38]. Virion proteomics have been used extensively to analyze the composition of a variety of virions, leading to a more complete picture of the viral particle.

However, to the best of our knowledge there is no mention of incorporation of host proteins in the enveloped-virus NDV so far. In this study, we selected the widely used NDV vaccine strain La Sota, utilized 2-DE/MS approaches to conduct a comprehensive proteomic analysis of purified NDV particles. Our analysis resulted in the identification of five virus-encoded structural proteins and thirty incorporated host proteins. Furthermore, the presence of five selected cellular proteins in the purified NDV particles was verified by Western blot or immunogold labeling detection.

## Results

### Purification of NDV virions

Virion proteomic analysis requires large quantity of virions for preparation of highly purified virus particles. Therefore, the choice of host system used for virus growth is an important consideration. Since specific pathogen free (SPF) embryonated chicken eggs are the preferred host system for growth of NDV and the chicken genome is already well annotated which would benefit the identification of cellular proteins, the 9-day-old SPF embryonated chicken eggs were selected as the host system for NDV propagation in this study.

The allantoic fluid (AF) with enrichment of NDV virions harvested at 108 h post-infection was clarified by differential centrifugation in order to remove the contamination of nuclei, mitochondria, lysosomes, peroxisomes from the chicken embryo. The virus was concentrated and firstly purified through a 20% (W/V) sucrose cushion before further purified over a non-linear 20%-60% sucrose-TNE (Tris-buffered saline including 50 mM Tris, 100 mM NaCl, 1 mM EDTA, pH 7.4) gradient. The high density opalescent virus band was observed at 40%–50% sucrose-TNE gradients.

**Figure 1:**
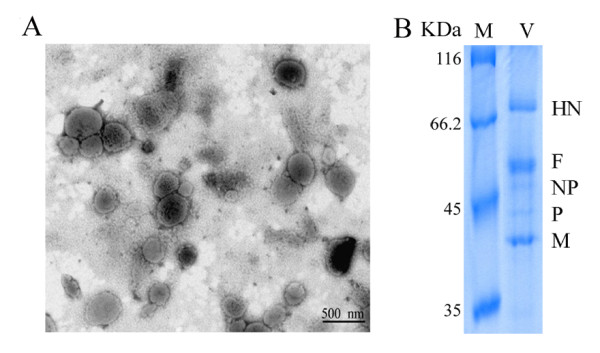
**Analysis of purified NDV particles. **(**A**) Electron micrograph of negatively stained with 2% potassium phosphotungstate (pH 6.5), sucrose density gradients purified NDV La Sota virus at 50,000 × magnification. (**B**) SDS-PAGE separation of proteins in purified NDV particles. 15 μg total proteins were separated on a 5-15% polyacrylamide gel and stained with coomassie blue. The positions of the viral proteins identified by their predicted molecular weights were indicated. M: protein marker, V: purified NDV, HN: Haemagglutinin Neuraminidase, F: Fusion protein, NP: Nucleocapsid protein, P: Phosphate protein, M: Matrix protein.

The purity of NDV La Sota was confirmed by electron microscopy analysis following negative staining. An abundance of intact virions was observed without obvious contamination from host cellular materials (Figure 1A). For further identification of the virions protein composition, the purified NDV particles were separated by sodium dodecylsulfate polyacrylamide gel electrophoresis (SDS-PAGE) and stained with coomassie brilliant blue (Figure 1B). Five major viral proteins (HN, F, M, NP and P) were evident but L protein was not visible. There were also some lighter bands visible which may represent cellular proteins incorporated into the NDV particles. Taken together, the highly purified NDV La Sota particles were obtained.

### Proteomic analysis of purified NDV particles

**Figure 2:**
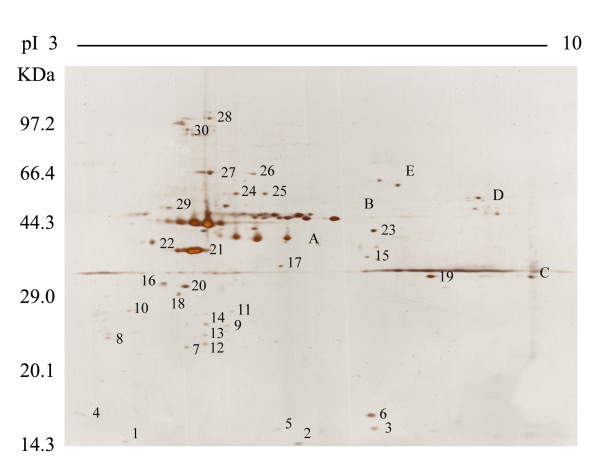
**Representative 2-DE gel images of purified NDV paricles. **Proteins (150 μg) were separated on the first dimensional pI 3–10 non linear IPG gels and second dimensional 5–15% continuous gradient vertical gels. Number 1–30 represent thirty host cellular proteins, A-E represent the structural proteins of NDV. The identified spots are numbered according to Table 1.

To obtain a detailed protein composition of NDV virions, viral proteins of purified NDV particles were extracted for 2-DE analysis with 150 μg of protein loaded on 18 cm gel strip (pI 3–10). To minimize inter-gel and inter-sample variation, three repeats of independent sample preparations and three repeats of independent 2-DE were performed under the identical conditions. After the electrophoresis separation, gels were stained with silver and processed for image analysis. A total of 45 protein spots were detected on the silver stained gel (Figure 2).

### Identification and functional classification of NDV-associated proteins

To identify the 45 protein spots obtained from the 2-DE separation, all protein spots were picked out from the stained gels, subjected to in-gel tryptic digestion and subsequently to identification by Matrix-assisted laser desorption/ionizationtime of flight mass spectrometry (MALDI-TOF/TOF) analysis. Database searching identified all NDV structural proteins except L protein, and revealed thirty cellular proteins as well. A complete list of all identified proteins with detailed information is shown in Table 1.

**Table 1:** Virus-encoded structural proteins and cellular proteins associated with purified NDV particles identified by MALDI-TOF/TOF MS

**Spot no.**^**a**^	**Protein description**^**b**^	**Protein Funtion**	**Accession** **no.**^**c**^	**Theoretical** **MW**^**d**^**/pI**^**e**^	**Score**^**f**^	**Pep.** **no.**^**g**^	**Reported in other viruses**
A	NDV NP	Viral protein	AAC28371	53047/5.29	455	19	
B	NDV P	Viral protein	AAC28372	42383/6.01	582	17	
C	NDV M	Viral protein	AAC28373	39538/9.58	149	8	
D	NDV F	Viral protein	AAC28374	58896/8.56	232	7	
E	NDV HN	Viral protein	AAC28376	63213/7.13	105	6	
1	S100 calcium-binding protein A6	Calcium ion binding	IPI00572547	10269.3/4.91	125	8	IBV[37]
2	S100 calcium-binding protein A11	Calcium ion binding	IPI00599279	11405.8/6.08	98	6	IV[35], VV[25,26], EBV[19], HCMV[17], KSHV[20,21], VSV[36], IBV[37]
3	aldolase A	Metabolic process	IPI00583507	14429.2/6.13	214	4	PRRSV[38]
4	Ras small GTPase	Signal transduction	IPI00683096	18292.1/4.64	76	7	
5	tumor protein, translationally-controlled 1	Calcium ion binding	IPI00821768	19517.6/4.9	95	3	
6	tumor protein D52	Calcium ion binding	IPI00579134	19848/4.94	114	5	
7	YWHAE 14-3-3, zeta polypeptide	Enzyme binding	IPI00820692	23944.1/5.23	211	6	IBV[37]
8	chromatin modifying protein 5	chromatin modifying	IPI00589508	24586.4/4.74	136	4	KSHV[39]
9	chromatin modifying protein 2A	chromatin modifying	IPI00594797	24716.8/5.57	158	5	KSHV[39]
10	chromatin modifying protein 4B	chromatin modifying	IPI00582041	25139.7/4.73	196	6	KSHV[39]
11	chromatin modifying protein 4 C	chromatin modifying	IPI00580681	25145.6/5.5	95	7	KSHV[39]
12	YWHAE 14-3-3, theta polypeptide	Enzyme binding	IPI00577739	27764.7/4.68	89	3	IBV[37]
13	nucleoporin 210kDaaa-like	Uncharactered	IPI00813608	28743.7/4.69	253	7	
14	YWHAE 14-3-3, epsilon polypeptide	Enzyme binding	IPI00579092	28944.4/4.75	162	4	IBV[37]
15	syntenin;syndecan binding protein	Signal transduction	IPI00598186	32037.6/7.01	187	6	
16	tropomyosin 1 alpha	cytoskeleton	IPI00600961	32482.6/4.68	93	5	HIV[31,32], IV[35]
17	capping protein muscle Z-line, alpha 2	cytoskeleton	IPI00683096	32964.7/6.34	86	7	HIV[31,32], IV[35]
18	Annexin A8-like 1	Uncharactered	IPI00585409	36709.9/5.24	153	10	
19	Annexin A2	cytoskeleton	IPI00577039	38615.9/6.92	172	12	HCMV[17], HIV[31,32], SARS [33], IV[35], IBV[37], PRRSV[38], herpes simplex virus 1[40]
20	KIAA0174	cytoskeleton	IPI00581393	39714.6/5.22	106	6	PRRSV[38]
21	Actin, gamma 1 propeptide;	cytoskeleton	IPI00572084	41808.8/5.3	453	12	HIV[31,32], SARS [33,34], IV[35], VV[25,26], EBV[19], HCMV[17], KSHV[20,21], VSV[36], IBV[37], PRRSV[38]
22	ovalbumin	Metabolic process	IPI00583974	42853.5/5.19	269	10	IBV[37]
23	ARP2 Actin -related protein 2 homolog	Uncharactered	IPI00585509	44673.3/6.3	219	8	
24	ARP3 Actin -related protein 3	cytoskeleton	IPI00587398	47391.1/5.62	206	7	IBV[37]
25	similar to type I hair keratin KA31	Uncharactered	IPI00587107	51130.7/4.78	91	4	
26	Ezrin	cytoskeleton	IPI00578484	69323.9/5.9	135	6	HIV[31,32], IV[35]
27	Heat shock protein 70	Molecular chaperone	IPI00818704	70955.3/5.43	140	4	HIV[31,32], SARS [33,34], IV[35], VSV[36], IBV[37], PRRSV[38], Adenovirus[41], enterovirus[42], vaccinia virus[43], hantaan virus[44]
28	CAP-GLY containing linker protein 2	cytoskeleton	IPI00579643	116233.3/6.45	81	3	IBV[37]
29	Tubulin, alpha-1	cytoskeleton	IPI00591483	49639/4.78	259	6	HIV[31,32], SARS [33,34], IV[35], VV[25,26], EBV[19], HCMV[17], KSHV[20,21], VSV[36], IBV[37], PRRSV[38]
30	heat shock 90 kDa aa protein 1, alpha	Molecular chaperone	IPI00596586	84006.5/5.01	130	7	IBV[37]

a Protein spot numbers on 2-DE gel.

b Alternative names are provided in parentheses.

c Accession numbers ford NCBI database (accessible at http://www.ncbi.nlm.nih.gov/).

d Theoretical molecular mass.

e Theoretical pI.

f MASCOT protein score (based on combined MS and MS/MS spectra) of greater than 64 (p ≤ 0.05) or the total ion score (based on MS/MS spectra) of greater than 30 (p ≤ 0.05).

g Observed peptides that differ either by sequence, modification or charge.

To better understand the implications of cellular proteins identified in NDV particles, these proteins were functionally categorized with biological processes according to Uniprot knowledgebase (Swiss-Prot/TrEMBL) and Gene Ontology Database. The identified thirty cellular proteins were composed of nine cytoskeletal proteins, two molecular chaperones, four chromatin modifying proteins, three enzymes-binding proteins, two calcium-binding proteins, two metabolism proteins, two signal transduction-associated proteins, two tumor-associated proteins (also characterized as calcium-binding proteins) and four uncharacterized proteins (Table 1). We firstly identified two signal transduction-associated proteins (syntenin and Ras small GTPase) and one tumor-associated protein (tumor protein D52) from the purified NDV particles.

### Validation of cellular proteins by western blot

**Figure 3:**
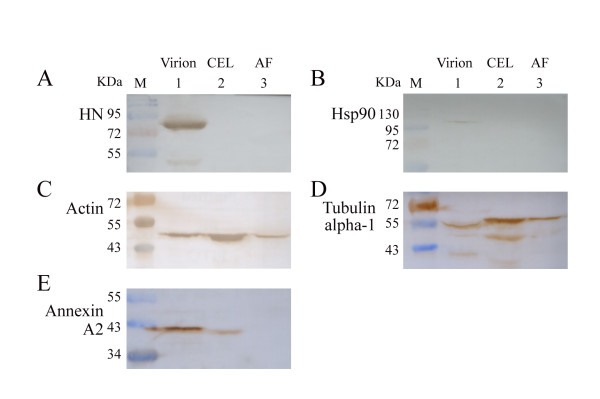
**Confirmation of host proteins incorporated into purified NDV particles by Western blot. **8 μg of purified virions from allantoic fluid (AF) (lane 1) and 15 μg of proteins extracted either from the normal 13-day-old specific pathogen free (SPF) chicken embryo (CEL) (lane 2) or from the AF of the uninfected 13-day-old specific pathogen free (SPF) chicken embryo eggs (lane 3) were subjected to western blot analysis with antibodies against the following proteins: (**A**) HN, (**B**) Hsp90, (**C**) Actin, (**D**) Tubulin alpha-1, (**E**) Annexin A2. Numbers to the left are molecular weight markers (M).

Western blot analysis was carried out for confirming the presence of host proteins associated with purified NDV particles. The proteins extracted from normal 13-day-old SPF embryonated eggs were included as a positive control; for negative control, AF from 13-day-old SPF embryonated eggs were performed with the same protocol as the purification of NDV virions. As shown in Figure 3, HN protein was only identified in purified NDV particles. HSP90, with low abundance in unstressed cells, was only detected associated with purified particles but not in cell extracts from 13-day-old SPF embryonated eggs. Actin, tubulin and annexin A2 were both found in the purified particles and positive control. It was expected that we also detected actin and tubulin in the AF extracts from uninfected SPF embryonated eggs, which resulted from their high concentrations in all eukaryotic cells and subcellular fractions.

### Validation of cellular proteins by electron microscopy and immunogold labeling

**Figure 4:**
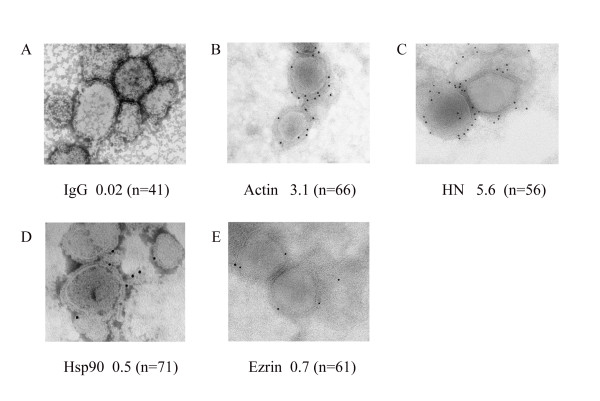
**Immunogold labeling of host proteins in purified NDV particles.** High purified NDV La Sota particles were immunogold labeled with antibodies against (**A**) normal mouse IgG, (**B**) Actin, (**C**) HN, (**D**) Hsp90, (**E**) Ezrin. The labeled virus were then negatively stained by 2% sodium phosphotungstate and visualized by electron microscopy (30,000× magnifications). Numbers shown outside the brackets indicate the quantity of gold particles per virion while numbers shown inside the blanket indicate the quantity of virions counted.

To provide additional evidence for the incorporation of host cellular proteins in NDV virions, immunogold labeling was performed. To remove the microvesicles from NDV virions, virions were subjected to digestion with bromelain and were then incubated with antibodies against HN protein of NDV La Sota strain, Actin, HSP90, Ezrin and normal mouse IgG with a gold-conjugated secondary antibody followed by negative staining (Figure 4). A couple of gold particles with HSP90 and Ezrin staining were found on the surface of a virion, which was significantly less than Actin and HN labeling. The result was consistent with the fact that there is far more HN present on the virions than HSP90 and ezrin. In addition, the abundance of Actin detected in the 2-DE gels is much higher than that of HSP90 and ezrin. These results indicated that the number of gold particles was consistent with the protein abundance in the gels.

## Discussion

There is compelling evidence that enveloped virions carry multiple host proteins both internally and externally during infection [15]. To date, no studies have been carried out on the incorporation of cellular proteins in NDV virions. In this study, we obtained highly purified NDV particles by sucrose gradients ultracentrifugation. Virion-associated proteins were identified by 2-DE/MS proteomic analysis followed by Western blot and electron microscopy. A total of five viral proteins and thirty host proteins were successfully detected. Our study provided strong evidence that cellular proteins were incorporated into the enveloped viruses.

The present study identified all the structural constitutes of NDV virions except L protein. F and HN are two major glycosidoproteins located on the surface of membrane, which are easy to detect in intact virions. M protein is also easy to identify because it is the most abundant structural protein produced throughout the process of the virus infection. The RNP complex of NDV particle contains three inner protein components, the major structural subunit (NP) and two associated proteins (P and L) binding together to RNA genome. Both P and NP protein were successfully obtained due to their comparatively higher expression level and smaller molecular weight; whereas the identification of L protein by MS was a difficult task, possibly due to its low-abundant in the virion and large molecular weight (about 220 kD).

Of the thirty host cellular proteins associated with purified NDV particles we have identified, a significant number of proteins have also been reported to be present in virions of other virus families, such as herpes viruses, poxviruses and retroviruses [14-16]. Considering that these studies were performed independently using different cell types and different mass spectrometry methods, this similarity is probably not an issue of contamination. The most likely explanation is that these viruses all share some fundamental feature and that these host proteins are involved in the processes associated with that common trait. Enveloped viruses enter the cell via a membrane fusion manner and exit by budding. Therefore, one hypothesis would be that these common incorporated host proteins play a role in the entry and release stages of the virus life cycle.

Enveloped viruses acquire their membranes through budding from the host cell, thus cytoskeleton proteins may be integrated inside the virions because of their propinquity to viral assembly and budding sites. Our virion proteomics identified 9 host cytoskeleton system proteins in purified NDV particles, which were the most abundant group of cellular proteins, including Tropomyosin 1 alpha, actin, actin -related protein 3 ARP3, tubulin alpha-1, annexin A2, ezrin, CAP-GLY domain containing linker protein 2, capping protein (Actin filament) muscle Z-line and KIAA0174. Numerous viral proteins interact with cytoskeleton elements. Available evidences indicate that host cytoplasm cytoskeleton components are involved in virus transport in cells, especially in the stages of virus entry and release [45]. Several studies have also indicated that cytoskeleton proteins such as Tubulin and Actin are required for viral gene expression [46,47] and are involved in several virus budding processes [48]. Interestingly, actin was originally thought as a cellular contaminant, but later demonstrated to be an internal component of the measles virus [49,50]. In a number of viruses, such as HIV and moloney murine leukemia virus (MMLV), actin is important during their budding [51-53]. For influenza virus, actin plays indispensable roles during the endocytosis of the virus into polarized epithelia [54]. An association of M with cytoskeleton elements has been reported [55], which indicates an essential function of actin in the replication cycle of coronavirus IBV. As for NDV, early studies have suggested that the cellular cytoskeletal framework actively participated in the structural and functional assembly of NDV transcriptive complex [56]. Therefore, cytoplasm cytoskeleton-associated proteins might take part in the assembly and budding process of newly formed NDV virions, contribute to the transportation of the virus to the correct location of host cell, and also participate in assembling the RNP complex.

Annexins are a well-known multigene family of Ca^2+^ regulated phospholipid-binding and membrane binding proteins with diverse functions. The presence of annexin A2 is thought to support viral binding, fusion and replication [57-61]. In the present study, cellular annexin A2 was also identified in purified NDV virions, which has been found to be endogenously associated with HCMV, HIV, IV virions, and herpes simplex virus 1 [31,35,40,62]. The exact role of annexin A2 in NDV life cycle needs to be further investigated.

Heat-shock proteins (HSP), known as molecular chaperones, has been identified in a number of envelope viruses. Several viruses require host molecular chaperones for entry, replication, and assembly, as well as other steps in viral production [63,64]. In this study, we identified two molecular chaperones incorporated into purified NDV particles, HSP70 and HSP90. HSP70 interacts with various viral proteins and may be involved in the assembly of adenovirus [41], enterovirus [42], vaccinia virus [43] and hantaan virus [44]. Virion-associated HSP70 might participate in early events of infection, uncoating the viral capsid in a manner similar to its role in the uncoating of clathrin cages[65]. HSP70 and HSP90 have been shown to interact with hepatitis B virus reverse transcriptase and to facilitate the initiation of viral DNA synthesis from hepatitis B virus pregenomic RNA [66,67]. HSP90, which can cooperate with other proteins such as p23 and HSP70, has 2–4 copies existing internally in a duck liver virus particle, and might be related to interaction between virus polymerase [68]. Besides, it has been proposed that HSP90 is a major host factor for viral replication of many RNA viruses [69], implying a important role of HSP90 in NDV replication.

In this study, NDV virion proteomic analysis revealed four chromatin modifying proteins, including chromatin modifying protein 2A, 4B, 4 C and 5. It was reported that they can be expressed in chicken bursal lymphocytes, and may be associated with regulating a variety of gene expression in lymphocytes [70]. Chromatin modifying proteins have also been found in KSHV by virion proteomics [39], providing a number of clues and potential links to understanding the mechanisms regulating the replication, transcription, and genome maintenance of KSHV. Therefore, NDV virion might modulate the gene expression of host cells through binding with chromatin modifying proteins for better propagation.

According to our investigation, three enzymes-binding proteins were identified, including tyrosine 3-monooxygenase/tryptophan 5-monooxygenase activation protein, zeta polypeptide; epsilon polypeptide and theta polypeptide, which have also been found expressed in chicken bursal lymphocytes [70], and may be related to the metabolic pathways during embryo development [71]. Meanwhile, two calcium-binding proteins, calcium-binding protein A6 and A11 were associated with the purified NDV particles. The calcium-binding proteins play a vital role in the regulation of cellular growth and signal transduction pathways; however, their effect on virus infection remains to be investigated [31,35,38].

Among the indentified cellular proteins in our study, three have not yet been reported in other viruses, including two signal transduction-associated proteins (syntenin and Ras small GTPase) and one tumor-associated protein (tumor protein D52), which have not been described to be present in other virions of quite diverse virus families. Previous work has identified syntenin of the shrimp Penaeus monodon (Pm) as a dynamic responder to white spot syndrome virus (WSSV) infection through its interaction with alpha-2-macroglobulin (alpha2M), which plays an important role in the immune defense mechanisms of viral infections of shrimps [72]. Ras small GTPase is a very important host signaling mediator, regulating the replication of viruses [73,74]. The molecular mechanisms of these two signaling mediators are largely unknown.

## Conclusions

Our virion proteomic analysis of purified NDV particles revealed the presence of five viral structural proteins and successfully identified thirty incorporated cellular proteins. It is reasonable to speculate that the incorporated cellular proteins in NDV virions may play roles in virus replication and virulence. Future experiments involving RNAi knockdown of these host proteins coding genes will help to address these questions. Indeed, a better understanding of cellular proteins in NDV virions may provide novel targets for the design of antiviral drugs as well as vaccines.

## Methods

### Propagation and purification of NDV

NDV La Sota strain (Beijing Merial Vital Laboratory Animal Technology Co, Ltd, Beijing, China) were propagated in 9-day-old specific pathogen free (SPF) embryonated eggs (Beijing Merial Vital Laboratory Animal Technology Co, Ltd, Beijing, China) at 37°C. The allantoic fluid (AF) with enrichment of NDV virions harvested at 108 h post-infection was clarified by differential centrifugation at 4°C, first centrifugated at 4,000 × g for 15 min and then the supernatant was centrifugated at 12,000 × g for 30 min. The viral supernatant was concentrated and firstly purified at 31,000 rpm through 5.5 ml of 20% (W/V) sucrose in TNE buffer (50 mM Tris, 100 mM NaCl, 1 mM EDTA, pH 7.4) for 2 h in a 70Ti rotor (Beckman Coulter, Optima™ L-100XP Preparative ultracentrifuge) at 4°C. Condensed and firstly purified virus pellet was then resuspended in TNE buffer and loaded on a preformed sucrose density gradient (20%, 30%, 40%, 50%, and 60% W/V) in TNE buffer for further purification. After centrifugation at 24,100 rpm for 2 h at 4°C in a SW41 rotor (Beckman Coulter, Optima™ L-100XP Preparative ultracentrifuge), the purified virus band between 40%-50% sucrose gradient was collected, diluted in approximately 1 ml of TNE buffer, and finally centrifuged at 24,100 rpm for 2 h at 4°C in a SW41 rotor to exclude the residuary sucrose. In order to get high purified NDV particles, the collected banded viruses were purified for a second time according to the same purification procedure. The purified virus pellet was stored at −80°C for further use.

### Validation of purified NDV particles by electron microscope and SDS-PAGE

Highly purified virus (3 μl) was adsorbed to Formavar-supported, carbon-coated nickel grids (230 mesh) for 2 min at room temperature (RT). The grids were negatively stained with 2% phosphotungstic acid and examined under a JEM-1400 electron microscope (JEM-100CX-II, JEOLLTD, Japan) operated at 120 kV.

Sodium dodecyl sulfate-polyacrylamide gel electrophoresis (SDS-PAGE) was also performed to validate the purified NDV particles. Proteins from the purified virus (15 μg) were denatured at 100°C for 10 min in 1× (SDS-PAGE) sample buffer and were then separated by SDS-PAGE. Coomassie Blue R250 was used for protein staining.

### Two-dimensional gel electrophoresis (2-DE) separation of proteins of purified NDV particles

The purified NDV particles were dissolved in 500 μl virus lysis buffer (7 M Urea, 2 M thiourea, 2% Triton X-100, 100 mM DTT, 0.2% IPG buffer pH 3–10) and incubated at 4°C for 1 h. After lysing by sonication (pulse durations of 2 s on and 3 s off) in an ice bath for 5 min, the lysates were clarified by centrifugation at 12,000 × g for 30 min at 4°C. The supernatant was collected and the concentration was determined by 2-DE Quant kit (Amersham, USA). The viral protein samples were then aliquoted and stored at −80°C for further analysis.

The first-dimension separation was performed using 18 cm Ready Strip IPG strips (non-linear, pI 3–10, GE Healthcare) for isoelectric focusing (IEF). The IPG strips were rehydrated with 400 μl rehydration buffer (7 M urea, 2 M thiourea, 2% (w/v) CHAPS, 65 mM DTT, 0.2% IPG buffer pH 3–10) containing 150 μg protein for 12 h at 20°C by a passive rehydration method. IEF was carried out at 20°C on an Ettan IPGphor III electrophoresis unit (GE Healthcare), and performed as follows: 100 V, linear, 100 Volt-Hours (Vhs); 200 V, Gradient, 200 Vhs; 500 V, linear, 500 Vhs; 1,000 V, linear, 1000 Vhs; 4,000 V, Gradient, 4,000 Vhs; 8,000 V, linear, 32,000 Vhs. The IPG strips were incubated for 15 min with gentle shaking in an equilibration buffer (6 M urea, 30% glycerol, 2% SDS and 0.375 M Tris–HCl, pH 8.8) with 1% (w/v) DL-Dithiothreitol (DTT) followed by additional equilibration for 15 min in SDS equilibration buffer containing 2.5% iodoacetamide (IAA).

The second-dimensional separation was carried out by using 5%-15% continuous gradient SDS-PAGE in Tris: glycine buffer (192 mM glycine, 25 mM Tris, 0.1% SDS, pH 8.3) at 140 V for about 10 h. The gels were stained by the modified silver staining method compatible with MS [75] and scanned at a resolution of 600 dpi using the Image scanner (Amersham Pharmacia Biotech). Spot detection, spot matching, and quantitative intensity analysis were performed using Image Master 2D Platinum 5.0 according to the manufacture’s protocol (GE Healthcare).

### In-gel tryptic digestion

The protein spots on the silver-stained gels were excised and transferred into 0.5 ml Eppendorf tubes, washed three times with ddH_2_O, destained with 15 mM potassium ferricyanide (K_3_Fe(CN)_6_, Amresco) and 50 mM sodium thiosulfate (NaS_2_O_3_, Amresco) in 50 mM NH_4_HCO_3_. After hydrating with 100% acetonitrile (ACN, Wako) and drying in a SpeedVac concentrator (Thermo Savant, USA) for 20 min, the gels were incubated with 12.5 ng/μl trypsin (Sequenceing grade, Promega) at 37°C overnight. The supernatant was collected and transferred into a 200 μl microcentrifuge tube, while the gels were extracted once with extraction buffer (67% ACN containing 5% trifluoroacetic acid (TFA, Wako)) at 37°C for 1 h. The supernatant of the gel spots were combined and then completely dried thoroughly in SpeedVac.

### MALDI-TOF/TOF MS, MS/MS analysis and database searching

Protein digestion extracts were resuspended with 5 μl of 0.1% TFA, and then the peptide samples were mixed (1:1) with a matrix consisting of a saturated solution of α-cyano-4-hydroxy-trans-cinnamic acid (α-CCA, Sigma) in 50% ACN containing 0.1% TFA. Digested proteins (0.8 μl) of each sample were spotted onto stainless steel target plates and allowed to air-dry at RT. Peptide mass spectra were obtained on an Applied Biosystem Sciex 4800 MALDI-TOF/TOF Plus mass spectrometer (Applied Biosystems, Foster City, CA). Data were acquired in positive MS reflector using a CalMix5 standard to calibrate the instrument (ABI 4800 Calibration Mixture). Mass spectra were obtained from each sample spot by accumulation of 900 laser shots in an 800–3500 mass range. For MS/MS spectra, the 5–10 most abundant precursor ions per sample were selected for subsequent fragmentation and 1200 laser shots were accumulated per precursor ion.

Combined MS and MS/MS spectra were submitted to MASCOT searching engine (V2.1, Matrix Science, London, UK) by GPS Explorer software (V3.6, Applied Biosystems) for proteins identification. Parameters for searches were as follows: trypsin as the digestion enzyme, one missed cleavage site, partial modification of cysteine carboamidomethylated and methionine oxidized, none fixed modifications, MS tolerance of 60 ppm, MS/MS tolerance of 0.25 Da. MASCOT protein score in IPI_CHICKEN (V3.49) database (based on combined MS and MS/MS spectra) of greater than 57 (p ≤ 0.05) or in NCBInr database of greater than 67 (p ≤ 0.05) was accepted.

### Validation of cellular proteins by western blot

Mouse monoclonal antibodies against actin (MAB1501), HSP90 (05–594) and NDV (HN14f) were purchased from Millipore. Rabbit polyclonal antibodies against annexin A2 (ab40943) and tubulin alpha-1 (ab4074) were products of Abcam Corparation. The critical challenge of virion proteomics was to prove that the host proteins were really an integral part of the virions and are not just non-specifically attached to the outside of the virions or derived from the contaminants. To address this question, we performed control experiment. Extracts from 13-day-old SPF embryonated eggs were designed as a positive control; AF from 13-day-old SPF embryonated eggs performed with the same protocol as the purification of NDV virions was used as a negative control.

The highly purified NDV particles were suspended in 1 × loading buffer (50 mM Tris–HCl pH 6.8, 2% SDS, 0.1% bromophenol blue, 10% glycerol, 100 mM DTT) and denatured by heating at 100°C for 5 min. The viral protein samples were then separated at 120 V on linear 5%-15% SDS-PAGE with 5% stacking gels in Tris: glycine buffer for about 3 h. After separated by SDS-PAGE, the viral proteins were transferred onto a polyvinylidene fluoride membrane (PVDF, P/N 66485, BioTrace, Pall Corporation). The membrane was blocked in freshly prepared 5% bovine serum albumin (BSA) with 0.05% Tween-20 for 2 h at RT with constant agitation. The PVDF membrane was washed three times with Tris buffered saline buffer (TBS) plus 0.2% Tween-20 and incubated with properly diluted primary antibodies for 2 h at RT. Following three washes with TBS, the secondary antibody conjugated to horseradish peroxidase (HRP) (00001–14, Proteintech Group, Inc) was added for 1 h at RT. The chemiluminescence system (AR1022, Boster Bio-Technology Co. LTD) was used for detection of antibody-antigen complexes.

### Protease treatment of NDV virions

Purified virus particles equivalent to 50 μg protein was incubated with bromelain (BB0243, BBI) at 0.2 mg/ml in 50 mM DTT (pH 7.2) in Dulbecco’s phosphate buffered saline (PBS) at 37°C for 15 min. After incubation, the samples were directly centrifuged to equilibrium in 11.5 ml non-linear 20%-60% sucrose-TNE gradients at 24,100 rpm for 2 h at 4°C in a SW41 rotor (Beckman Coulter, Optima™ L-100XP Preparative ultracentrifuge). Condensed virus was diluted with TNE buffer, followed by sedimentation at 24,100 rpm for 2 h at 4°C in a SW41 rotor to remove the sucrose and were then subjected to immunogold labeling and electron microscopy analysis.

### Validation of cellular proteins by electron microscopy and immunogold labeling

Rabbit polyclonal antibody against chicken IgG (15 nm Gold) (ab41500), goat polyclonal against rabbit IgG (5 nm Gold) (ab27235) and goat polyclonal against mouse IgG (10 nm Gold) (ab27241) were purchased from Abcam. Protease treated NDV particles were suspended in PBS (pH 7.4) and then were collected onto 230-mesh formwar-coated nickel grids and adsorbed on the grids for 5 min. The virus particles were fixed in 2% paraformaldehyde for 5 min at RT, after treating with Triton X-100 (0.2%) in PBS (pH 7.4) for 5 min; the sample was blocked with 5% BSA in PBS-Tween 20 (pH 7.4) for 30 min at RT. All grids were then blocked with blocking buffer (5% BSA, 5% normal serum, 0.1% cold water skin gelatin, 10 mM phosphate buffer, 150 mM NaCl, pH 7.4) for 30 min. After washing with PBS, immobilized virions were incubated for 1.5 h with 50 μg/ml primary antibody (in 1% BSA), and washed three times for 5 min in PBS/1% BSA. Anti-rabbit or anti-mouse immunoglobulin G coupled to 10 nm colloidal gold particles was used as the secondary antibody and virions were incubated in it for 40 min at RT. The unbound antibodies were removed, the grids were thoroughly washed and negatively stained with 2% sodium phosphotungstate (pH 6.5) for 1 min. Negatively stained virions were examined on a scan and transmission electron microscope.

## Abbreviations

ND, Newcastle disease; NDV, Newcastle disease virus; 2-DE, Two-dimensional electrophoresis; SDS-PAGE, Sodium dodecylsulfate polyacrylamide gel electrophoresis; MS, Mass spectrometry; MALDI-TOF, Matrix-assisted laser desorption/ionization time of flight mass spectrometry; SPF, Specific pathogen free; AF, Allantoic fluid; BSA, Bovine serum albumin; IEF, Isoelctric focusing; DTT, Dithiothreitol; IAA, Iodoacetamide; ACN, Acetonitrile; TFA, Trifluoroacetic acid; TNE, Tris-buffered saline including 50 mM Tris, 100 mM NaCl, 1 mM EDTA, pH 7.4; PBS, Phosphate-buffered saline; TBS, Tris-buffered saline; HRP, Horseradish peroxidase; pI, Isoelectric point; MW, Molecular weight; RT, Room temperature; L, Litre; mL, millilitre.

## Competing interests

The authors declare no competing interests.

## Authors’ contributions

XR performed the main proteomic experiments and data analysis and drafted the manuscript. CX created the detailed experimental design. QK and CZ contributed to the initial phase of the proteomic experiments. YB and YC conceived study, and participated in its design, coordination and helped to sketch the manuscript. All authors have read and approved the final manuscript.
